# Clinical characteristics, treatment, and outcomes of fungal keratitis/endophthalmitis caused by *Purpureocillium lilacinum*

**DOI:** 10.3389/fcimb.2026.1805428

**Published:** 2026-06-01

**Authors:** Liuyang Hu, Kaining Long, Qianqian Lan, Li Jiang, Hui Huang, Xingchun Chen, Qi Chen

**Affiliations:** 1 Department of Laboratory Medicine, The People’s Hospital of Guangxi Zhuang Autonomous Region, Guangxi Academy of Medical Sciences, Nanning, China; 2 College of Life Science and Technology, Guangxi University, Nanning, China; 3 Department of Ophthalmology, The People’s Hospital of Guangxi Zhuang Autonomous Region, Guangxi Academy of Medical Sciences, Nanning, China; 4 Visual Science and Optometry Center, The People’s Hospital of Guangxi Zhuang Autonomous Region, Guangxi Academy of Medical Sciences, Nanning, China

**Keywords:** fungal keratitis, *in vitro* antifungal susceptibility test, ITS sequence, MALDI-TOF MS, *Purpureocillium lilacinum*

## Abstract

**Background:**

*Purpureocillium lilacinum* (*P. lilacinum*) is an emerging pathogenic fungus that can cause fungal keratitis and endophthalmitis, which pose a severe threat to patients’ visual acuity.

**Methods:**

A retrospective analysis was conducted on 20 cases (17 keratitis and 3 endophthalmitis) caused by *P. lilacinum*. Clinical manifestations, mycological findings, *in vitro* antifungal susceptibility, treatment regimens, and visual outcomes were evaluated.

**Results:**

A total of 20 patients with unilateral *P. lilacinum* ocular infections were enrolled. Among these 20 cases, 14 had a clear history of trauma, including 12 cases of plant-related injury and 2 cases of metallic injury. One case had a history of pepper powder exposure to the eye; one case had a history of long-term steroid eye drops after corneal transplantation; one case had allergic conjunctivitis with prolonged steroid eye drop use. No obvious predisposing cause was identified in 3 cases. Corneal scrapings/intraocular fluid or confocal microscopy revealed hyphae/spores, and fungal identification was confirmed by morphological analysis, matrix-assisted laser desorption/ionization time-of-flight mass spectrometry (MALDI-TOF MS), and internal transcribed spacer (ITS) sequencing. Antifungal susceptibility testing showed high minimum inhibitory concentrations (MICs) to amphotericin B, 5-flucytosine and fluconazole; and high minimum effective concentrations (MECs) to micafungin and caspofungin, but low MICs to triazoles (voriconazole, isavuconazole, posaconazole, itraconazole). Individualized treatment regimens were administered to the 20 patients based on their conditions, including pars plana vitrectomy combined with voriconazole infusion/anterior chamber irrigation, corneal debridement combined with pharmacotherapy, intrastromal corneal injection, and conjunctival flap covering. All patients received supplementary oral voriconazole and voriconazole eye drops. The treatment course lasted 20–96 days. Except for 3 cases with hand motion (HM) visual acuity, the best-corrected visual acuity (BCVA) of the remaining cases ranged from 0.2 to 0.8 LogMAR (Logarithm of the Minimum Angle of Resolution), and the conditions of most patients were effectively controlled.

**Conclusion:**

*P. lilacinum* is an emerging fungal pathogen that exhibits intrinsic resistance to polyene antifungal agents (amphotericin B, natamycin), while being susceptible to triazole drugs. Early microbiological diagnosis, aggressive lesion debridement, and combined drug therapy are crucial. For progressive lesions, therapeutic keratoplasty may be required. This pathogen can pose a severe threat to visual acuity, highlighting the necessity of timely intervention.

## Introduction

Fungal keratitis is a vision-threatening infection characterized by stromal inflammation and leukocyte infiltration, often necessitating urgent intervention (Karsten et al., 2012). Corneal trauma, particularly from plant material, is the most common risk factor globally (Bharathi et al., 2007; Xie et al., 2006). While *Aspergillus* and *Fusarium* spp. remain the leading causes of keratomycosis (Liu et al., 2023; Xie et al., 2006), emerging pathogens such as *Acremonium*, *Cladosporium*, and *P. lilacinum* have increasingly been reported (Sahay et al., 2020)*. P. lilacinum* (formerly *Paecilomyces lilacinus*) is a filamentous fungus associated with severe ocular infections, including keratitis and destructive endophthalmitis, and due to its resistance to polyene and other traditional antifungal agents, treatment with these drugs often fails (Pastor and Guarro, 2006). Although rare, these infections can lead to irreversible vision loss or enucleation, underscoring the need for prompt diagnosis and tailored therapy.

This study retrospectively analyzed 17 cases of *P. lilacinum* keratitis and 3 cases of endophthalmitis treated at the People’s Hospital of Guangxi Zhuang Autonomous Region between January 1, 2019, and December 31, 2024. We describe the clinical presentations, risk factors, antifungal susceptibilities, treatment strategies, and outcomes to inform the early management of this emerging pathogen.

## Materials and methods

### Patient

This study was approved by the Ethics Committee of the People’s Hospital of Guangxi Zhuang Autonomous Region, with approval number KY-KJT-2024-163.

A retrospective review was conducted on 20 patients with confirmed *P. lilacinum* infection (17 cases of fungal keratitis and 3 cases of fungal endophthalmitis) between January 2019 and December 2024.

Definite diagnosis of *P. lilacinum* infection required fulfillment of all three criteria:

(1) Detection of fungal hyphae or spores in corneal scrapings/intraocular fluid, or evidence of stromal hyphae on *in vivo* confocal microscopy;
(2) Fungal culture positive, showing rapid growth and characteristic vinaceous-violet colonies on Potato dextrose agar (PDA);
(3) Species confirmation by ITS sequencing of ribosomal DNA.

### Inclusion and exclusion criteria

#### Inclusion criteria

(1) Clinical diagnosis of fungal keratitis or fungal endophthalmitis with typical manifestations, including corneal ulcer, feathery hyphal infiltrate, satellite lesions, hypopyon, or inflammatory signs in the anterior chamber/vitreous cavity.
(2) Laboratory confirmation of *P. lilacinum* infection from corneal scrapings, biopsy specimens, or intraocular fluid, based on morphological characteristics combined with ITS sequencing.
(3) Complete clinical, laboratory, treatment, and follow-up data available for analysis.
(4) Patients received standardized antifungal therapy, with or without adjuvant surgical intervention.
(5) Written informed consent was obtained from patients or their legal representatives.

#### Exclusion criteria

(1) Concurrent or mixed infection with other pathogens, including bacteria, viruses, or acanthamoeba.
(2) Fungal infection caused by other genera (e.g., *Fusarium*, *Aspergillus*, *Candida*).
(3) Non-infectious corneal disorders, such as exposure keratitis, neurotrophic keratitis, or immune-mediated keratitis.
(4) Incomplete clinical, microbiological, treatment, or follow-up data insufficient for reliable analysis.
(5) Failure to meet ethical requirements or lack of signed informed consent.

**Figure 1:**
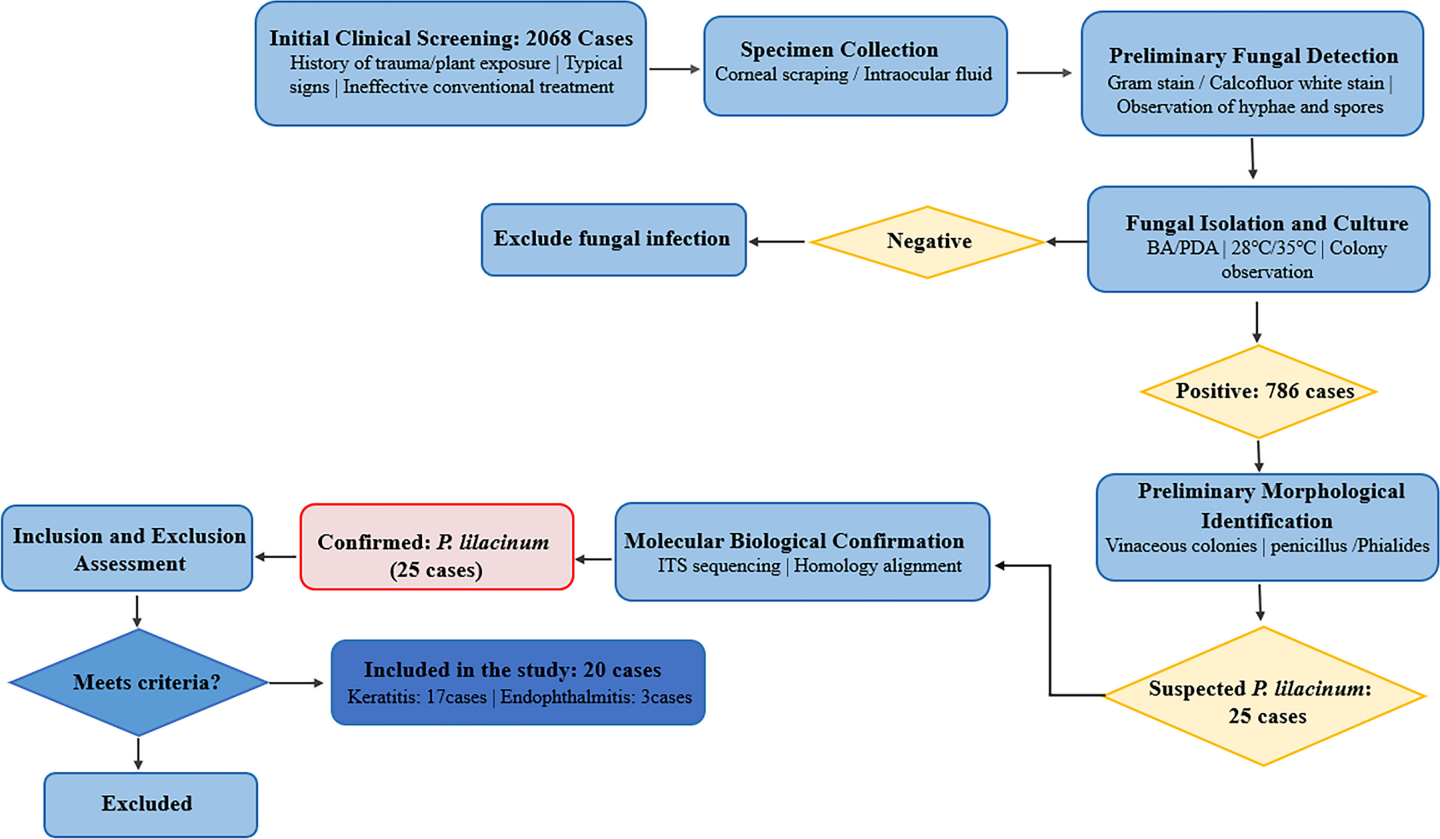
The flowchart of case inclusion in this study.

The flowchart of patient enrollment is illustrated in **Figure 1**.

Patient histories were collected, including presenting symptoms, risk factors for keratitis/endophthalmitis (such as plant-related injury, ocular surgery, and immunosuppression), time of onset, prior medication use, and disease course. BCVA and clinical ocular findings were documented. Hyphal morphology was evaluated by *in vivo* confocal microscopy. RTVue optical coherence tomography (OCT; Optovue, Fremont, CA, USA) was used to assess the depth of corneal ulceration.

### Microbiological culture and strain identification

Corneal scraping tissues or intraocular fluid were collected under sterile conditions and inoculated onto blood agar (BA) and PDA. Cultures were incubated at 35°C (BA) and 28°C (PDA) for up to 7 days. Colony morphology was observed, and isolates were stained with lactophenol cotton blue for microscopic examination.

### Strain identification and cluster analysis of MALDI-TOF ms

MALDI-TOF MS was used to identify the isolated strains. In an EP tube, 300 µL of deionized water was added, and one or two isolated colonies were picked with a moist swab into the centrifuge tube, thoroughly mixed. Then, 900 µL of ethanol was added and mixed well. The mixture was centrifuged at 13,000 rpm for 2 minutes, and the supernatant was discarded. A second centrifugation was performed, and residual supernatant was completely removed with a pipette, avoiding contact with the precipitate. The precipitate was dried at room temperature for 2–3 minutes, followed by the addition of 10 µL of 70% formic acid, which was vortexed to mix thoroughly. Then, 10 µL of acetonitrile was added, mixed well, and centrifuged at 13,000 rpm for 2 minutes. 1 µL of the supernatant was taken and spotted onto a MALDI target plate, dried at room temperature, then covered with 1 µL of HCCA matrix solution, and again dried at room temperature before being placed into the EXS2600 MALDI-TOF MS (Zybio Inc., Chongqing, China) for analysis. Spectra were analyzed using software Ex-accuspec V3. Species-level identification was confirmed with a score ≥2.0.

For cluster analysis, EX-Smartspec V1 generated a dendrogram using the complete linkage algorithm based on a cosine similarity matrix of the spectral peak profiles.

### ITS sequencing and phylogenetic analysis

Genomic DNA was extracted from isolates using the cetyltrimethylammonium bromide (CTAB) method. The ITS region was amplified with primers ITS1 (5’-TCCGTAGGTGAACCTGCGG-3’) and ITS4 (5’-TCCTCCGCTTATTGATATGC-3’). Amplicons were Sanger-sequenced at the Kingdom Clinical Trial Center (Guangzhou, China). Sequences were analyzed with Chromas 2.6.6 and compared against the NCBI GenBank and Westerdijk Fungal Biodiversity Institute databases. Phylogenetic trees were constructed using MEGA 7.0.26 via the Maximum Likelihood method with 1000 bootstrap replicates, including sequences of the reference strains of each species and outgroup species (information on the reference strains is provided in the Supplementary Material).

### Antimicrobial susceptibility testing

Antifungal susceptibility testing was performed according to Clinical Laboratory Standards Institute(CLSI) (2022). A colorimetric microdilution method was used with *Paecilomyces variotii* (ATCC MYA-3630) as the quality control strain. The panel included nine antifungal agents: amphotericin B, 5-flucytosine, micafungin, caspofungin, fluconazole, isavuconazole, voriconazole, posaconazole, and itraconazole (Zhuhai DL Biotech Co., Ltd, China).

Test strains were subcultured on PDA for 3 days, and suspensions were adjusted to 0.5 McFarland turbidity. Microdilution trays were incubated at 35°C in ambient air. MECs for micafungin and caspofungin were read at 21–26 hours, while MICs for other drugs were determined at 46–50 hours. MIC50 and MIC90 values were calculated based on cumulative data.

## Results

### Demographics and clinical characteristics

A total of 20 patients (13 males, 7 females; mean age 51.7 ± 12.3 years, range 28–82 years) were included, with 17 cases of keratitis and 3 cases of endophthalmitis. All 20 cases were unilateral. Of the 20 enrolled patients, 14 had a clear history of trauma, including 12 cases of plant-related injury and 2 cases of metallic injury. One case had a history of pepper powder exposure to the eye; one case had a history of long-term steroid eye drops after corneal transplantation; one case had allergic conjunctivitis with prolonged steroid eye drop use. No obvious predisposing cause was identified in 3 cases.

**Figure 2:**
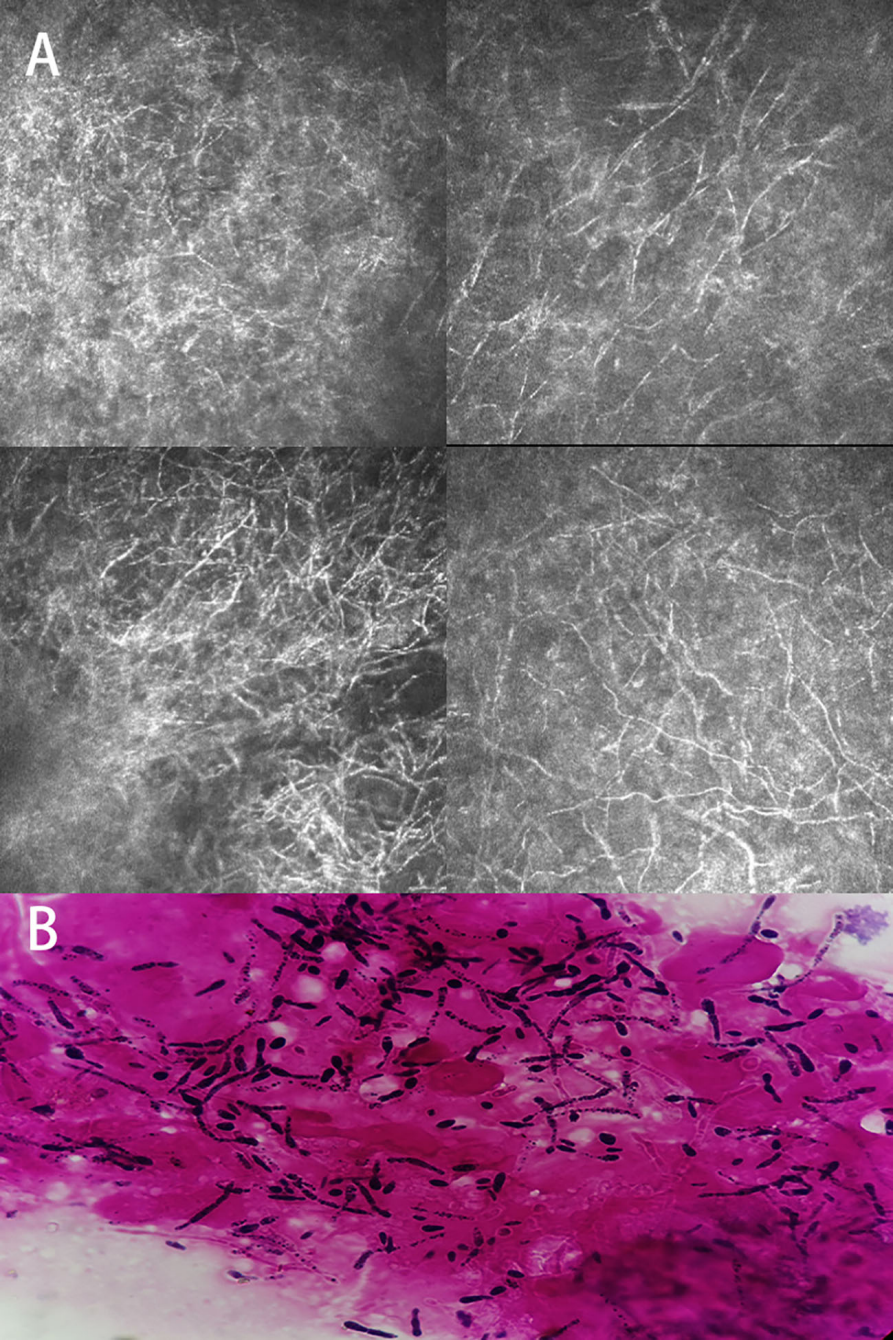
**(A)** Under confocal microscopy, the hyphae of *P. lilacinum* are thinner and more densely branched compared with those of the commonly seen *Fusarium* and *Aspergillus* species. However, due to high overlapping, it is difficult to distinguish them clearly, and the hyphae often present as a tangled mass; **(B)** gram staining revealed numerous spherical or oval conidia and hyphae; the conidia were mostly distributed singly, with a small number in clusters.

Among the 20 cases, 3 with endophthalmitis did not undergo confocal corneal microscopy examination. The remaining 17 cases were diagnosed as “corneal ulcer” at the initial visit and underwent confocal corneal microscopy. Among these 17 cases, hyphal-like structures were detected in the corneal tissue via the first confocal microscopy examination in 16 cases; only a large number of inflammatory cell structures were observed in the other 1 case. The positive detection rate of confocal microscopy reached 94.12%. Under confocal microscopy, the hyphae of *P. lilacinum* are thinner and more densely branched compared with those of the commonly seen *Fusarium* and *Aspergillus* species. However, due to high overlapping, it is difficult to distinguish them clearly, and the hyphae often present as a tangled mass. Meanwhile, around the infected lesions, some highly reflective, irregularly shaped, punctate or clustered inflammatory cells can usually be observed gathering around the hyphae (**Figure 2A**). All 20 cases were culture-positive for *P. lilacinum*, with sampling sites as follows: 17 cases were corneal scraping tissues, and 3 cases were intraocular fluid. In 16 cases, Gram staining revealed numerous spherical or oval conidia and hyphae; the conidia were mostly distributed singly, with a small number in clusters (**Figure 2B**).

Under slit-lamp microscopy, corneal ulcers caused epithelial defects and partial loss of the corneal stroma in the lesion area, presenting with feathery or villous edges. The boundary between the infiltrated and non-infiltrated areas was blurred, and the surface was covered with a toothpaste-like fungal mycelial plaque. In some cases, a ring-shaped infiltration was observed around the ulcer, separated from the central ulcerative area by a relatively transparent zone. The shallowest corneal ulcer reached only 1/4 of the corneal thickness (CT), while the deepest extended to full-thickness, with some cases accompanied by endothelial plaques. Among the 20 cases, 18 were complicated with anterior uveitis, characterized by the presence of keratic precipitates (KP) on the corneal endothelium and aqueous flare. Of these 18 cases, 13 developed hypopyon ranging from 1 to 3 mm in height. Excluding 2 eyes diagnosed with endophthalmitis without obvious corneal ulcers, 4 out of the remaining 18 eyes experienced corneal perforation. Among these 4 cases, 2 were caused by direct ocular trauma, with the perforation sites located exactly at the limbus. The other 2 cases of perforation resulted from progressive ulceration that was refractory to antifungal therapy.

### Mycology

**Figure 3:**
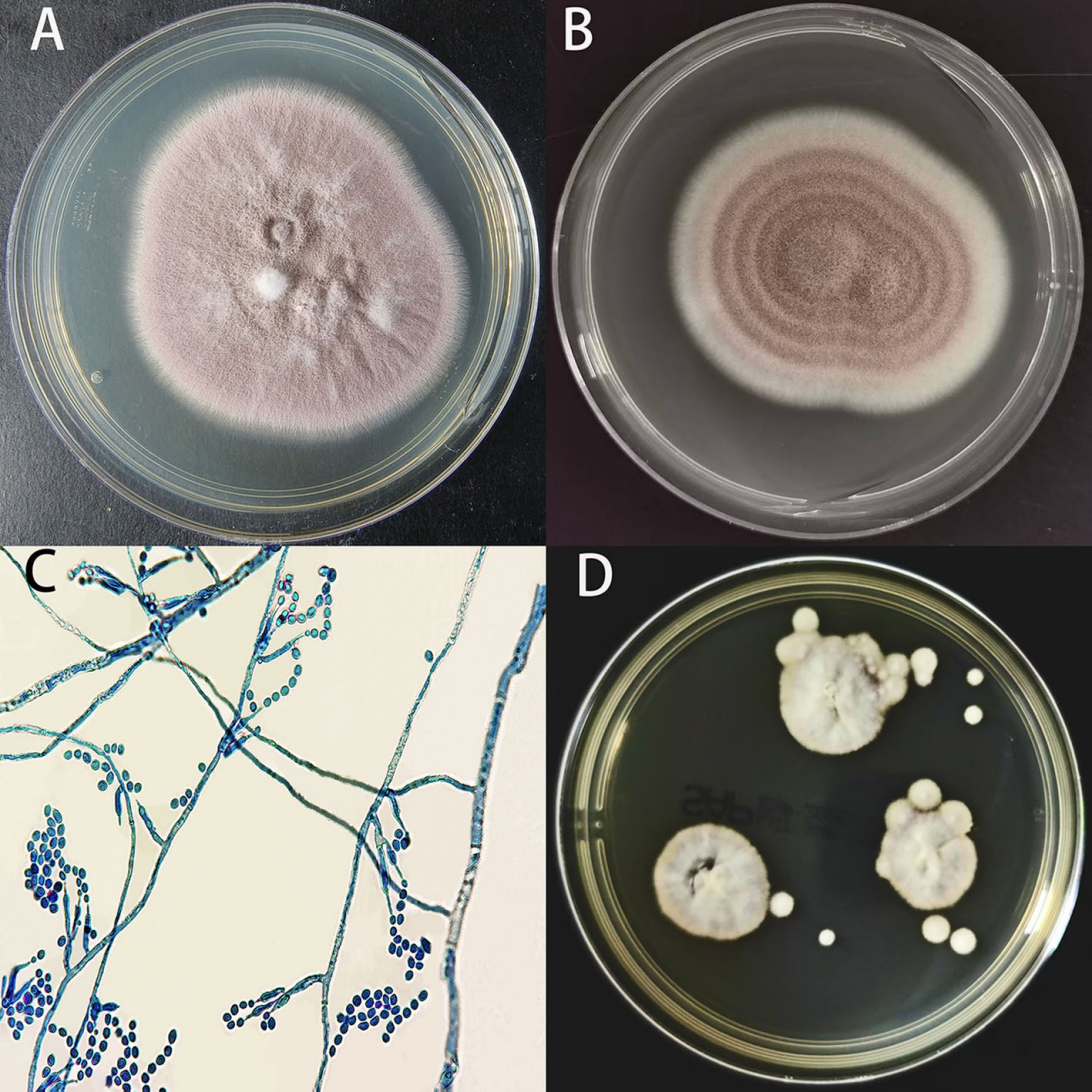
**(A)** dispersed fluffy colonies on PDA, 7 d, 28 °C; **(B)** concentric whorl-like colonies on PDA, 7 d, 28 °C; **(C)** microscopic characteristics of *P. lilacinum* stained with cotton blue; **(D)** colonies on PDA, 7 d, 35 °C.

From the 20 specimens submitted for testing, 20 strains of filamentous fungi were isolated. On PDA at 28 °C, the colonies grew rapidly, appearing suede-like to floccose, and exhibited two morphologies: one was dispersed and fluffy ((**Figure 3A**), and the other was concentrically whorled (**Figure 3B**). The aerial mycelium was initially white but turned various shades of vinaceous when sporulating. The conidiophores were upright, 400-600 μm in length, and bore branches densely clustered with phialides. The stipes of the conidiophores were 3-4 μm wide, ranged from yellow to purple in color, and had rough walls. The phialides were swollen at the base and tapered into a slender neck. The conidia were ellipsoidal to fusiform, with smooth to slightly rough walls, hyaline to purple in mass, 2.5-3.0 × 2.0-2.2 μm in size, and produced in divergent chains (**Figure 3C**). Chlamydospores were absent. This strain exhibited a faster growth rate at 28 °C than at 35 °C, with poor growth observed at 35°C (**Figure 3D**).

### Species identification

**Figure 4:**
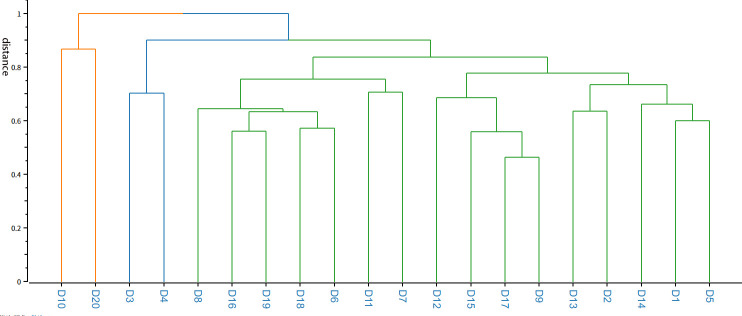
*P. lilacinum* hierarchical clustering of MALDI-TOF peak profiles identified two different clusters.

**Figure 5:**
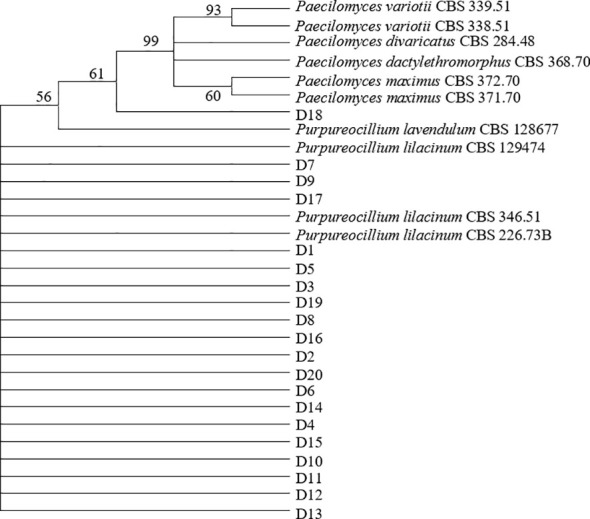
Maximum-likelihood phylogenetic tree based on the ITS sequences showing the relationship of isolated strains with type strains species. Numbers above the nodes represent bootstrapping values generated from 1000 replicates, using a Tamura 3-parameter+Gamma distributed model. Only values above 50% are indicated.

MALDI-TOF MS correctly identified 100% of the *P. lilacinum* isolates (identification scores ≥ 2.0). Hierarchical clustering of MALDI-TOF peak profiles identified two different clusters (**Figure 4**). ITS rDNA genes sequencing identified 20 isolates as *P. lilacinum*. Maximum-likelihood phylogenetic tree based on the ITS sequences showing the relationship of isolated strains with type strains species (**Figure 5**).

### Antimicrobial susceptibility testing

Determining MICs range for amphotericin B were 8 ~ >32µg/mL, MIC50 and MIC 90 were >32µg/mL; MICs for 5-fluorocytosine all >32 µg/mL; MICs range for fluconazole were16 ~ 64, and MIC50 and MIC 90 were 32µg/mL; MICs range for isavuconazole were 0.12 ~ 0.50, MIC50 and MIC 90 was 0.25µg/mL and 0.50µg/mL, respectively; MICs range for voriconazole were 0.06 ~ 0.25, MIC50 and MIC 90 was 0.12µg/mL and 0.25µg/mL, respectively; MICs range for posaconazole were 0.06 ~ 0.25, MIC50 and MIC 90 was 0.06µg/mL and 0.12µg/mL, respectively; MICs range for itraconazole were 0.12 ~ 1, MIC50 and MIC 90 was 0.25µg/mL and 0.50µg/mL, respectively. ALL MECs for micafungin and caspofungin were >8 µg/mL (**Table 1**). Up to now, no CLSI guideline protocol for epidemiological cutoff values and breakpoints in vitro susceptibility testing of *P. lilacinum* is available, except for the recommendation that AMB should be reported as “intrinsically resistant”. The results showed that all *P. lilacinum* isolates had high MICs or MECs to amphotericin B, 5-fluorocytosine, micafungin, caspofungin, and fluconazole; by contrast, isavuconazole, voriconazole, posaconazole, and itraconazole were highly active against *P. lilacinum*.

**Table 1:** Antifungal drug sensitivity of 20 strains of *P. lilacinum* isolated from the eye.

Antifungal drugs	Range (ug/mL)	MIC50/MEC50	MIC90/MEC90
AMB	8 ~ >32	>32	>32
5-FCT	>64	>64	>64
MIF	>8	>8	>8
CAS	>8	>8	>8
FLU	16 ~ 64	32	32
ISA	0.12 ~ 0.50	0.25	0.50
VOR	0.06 ~ 0.25	0.12	0.25
POS	0.06 ~ 0.25	0.06	0.12
ITR	0.12 ~ 1	0.25	0.50

### Treatment and outcomes

Different treatment regimens were adopted for 20 cases based on their respective conditions:

Regimen 1:

For 3 cases of endophthalmitis, 2 cases underwent pars plana vitrectomy combined with intravitreal injection of voriconazole (Pfizer) solution at a concentration of 100 μg/0.1 mL, and were additionally administered oral voriconazole (200 mg twice daily). One case with a large corneal ulcer and massive hypopyon received anterior chamber irrigation with voriconazole solution (100 μg/0.1 mL), followed by pus collection for culture. Postoperatively, the patients were prescribed oral voriconazole (200 mg twice daily) combined with 1% voriconazole eye drops administered hourly. Once the condition was controlled, the frequency of eye drop administration was reduced to 7 times per day. All 3 cases retained ocular integrity, with a final visual acuity of HM.

Regimen 2:

For 11 cases, corneal debridement and scraping were first performed, with the specimens sent for culture. Subsequently, the patients received oral voriconazole (Yangzijiang Pharmaceutical Group, 200 mg twice daily) combined with 1% voriconazole eye drops administered every 2 hours. Among these cases, corneal nebula or leukoma formed in 8 cases after ulcer control; 3 case underwent corneal transplantation due to treatment failure.

Regimen 3:

3 cases first underwent corneal debridement and scraping for culture. Thereafter, interlamellar corneal injection of 0.1% voriconazole solution was performed at 5–6 sites surrounding the corneal lesion (0.1 mL per injection site). Post-injection treatment included oral voriconazole (200 mg twice daily) combined with 1% voriconazole eye drops every 2 hours. The ulcers were effectively controlled in all 3 cases, with the formation of corneal nebula or leukoma as the final outcome.

**Figure 6:**
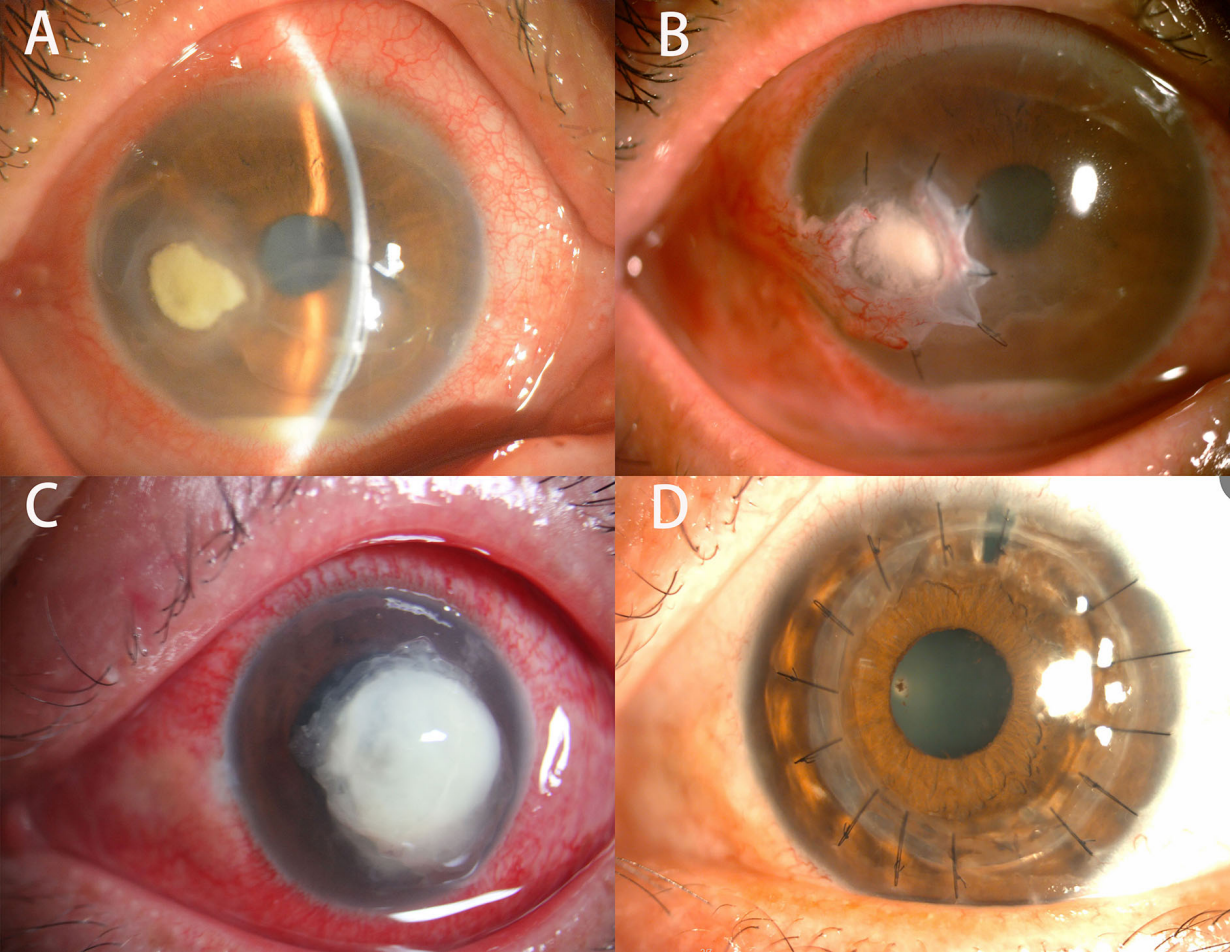
**(A)** a patient with *P. lilacinum* keratitis with uncontrollable condition before conjunctival flap covering; **(B)** the same patient after conjunctival flap covering; **(C)** uncontrollable corneal ulcer in a patient after treatment; **(D)** the same patient after corneal transplantation.

Regimen 4:

3 cases were initially managed with corneal debridement and scraping for culture, followed by conjunctival flap covering surgery to shield the corneal lesion. Postoperatively, the patients were treated with oral voriconazole (200 mg twice daily) combined with 1% voriconazole eye drops every 2 hours. The ulcers were controlled in 2 eyes, with subsequent development of corneal nebula or leukoma; 1 case underwent corneal transplantation due to progressive disease despite treatment (**Figure 6**).

The treatment duration of the 20 cases ranged from 20 to 96 days. At the end of the course, except for 3 cases with HM, the best-corrected visual acuity of the remaining cases was LogMAR visual acuity: 0.2 to 0.8. Details of 20 patients diagnosed with fungal keratitis/endophthalmitis caused by *P. lilacinum* are listed in **Table 2**.

**Table 2:** Details of 20 patients diagnosed as fungal keratitis/endophthalmitis caused by *P. lilacinum*.

Patient	Clinical diagnosis	Gender /age	Predisposing factor and risk factor	Depth of corneal ulcer (CT)	baseline BCVA	Treatment regimen	Treatment duration	Final BCVA	Follow-up duration	Outcomes	Complications
D1	Keratitis	Male /47	Post-corneal transplantation, long-term use of topical steroid eye drops	1/2	FC/30cm	Corneal debridement + oral voriconazole + voriconazole eye drops → failed to control → corneal transplantation	25d	0.2	6 months	Healed	No obvious complications
D2	Keratitis	Female /55	Plant-related injury	2/3	HM	Conjunctival flap covering+ oral voriconazole + voriconazole eye drops→ failed to control → corneal transplantation	23d	0.3	6 months	Healed	No obvious complications
D3	Keratitis	Male /33	Plant-related injury	Full-thickness	HM	Corneal debridement +oral voriconazole + voriconazole eye drops → failed to control → corneal transplantation	24d	0.7	6 months	Healed	No obvious complications
D4	Keratitis	Female /56	N	1/4	FC/30cm	Corneal debridement +oral voriconazole + voriconazole eye drops	20d	0.8	6 months	Healed	Corneal nebula
D5	Endophthalmitis	Female /41	Iron wire penetrating injury	Full-thickness	HM	Anterior chamber irrigation with voriconazole + oral voriconazole +voriconazole eye drops	96d	HM	6 months	Healed	Mild anterior chamber inflammation (resolved with medication)
D6	Keratitis	Female /49	Plant-related injury	1/4	0.4	Corneal debridement +oral voriconazole + voriconazole eye drops	26d	0.8	6 months	Healed	Corneal nebula
D7	Keratitis	Female /63	Plant-related injury	4/5	HM	Conjunctival flap covering+ oral voriconazole + voriconazole eye drops	36d	0.4	6 months	Healed	Corneal nebula
D8	Keratitis	Male /59	Screwdriver injury	Full-thickness	HM	Conjunctival flap covering+ oral voriconazole + voriconazole eye drops	52d	0.2	6 months	Healed	Corneal leukoma
D9	Endophthalmitis	Male /51	Plant-related injury	N	LP	Pars plana vitrectomy + intravitreal injection of voriconazole+ oral voriconazole	93d	HM	6 months	Healed	No obvious complications
D10	Keratitis	Male /51	Plant-related injury	1/3	FC	Corneal debridement +oral voriconazole + voriconazole eye drops	34d	0.4	6 months	Healed	Corneal leukoma
D11	Keratitis	Male /82	Plant-related injury	1/3	0.3	Corneal debridement +oral voriconazole + voriconazole eye drops	28d	0.8	6 months	Healed	Corneal leukoma
D12	Keratitis	Male /48	Plant-related injury	1/2	HM	Corneal debridement +oral voriconazole + voriconazole eye drops→ failed to control → corneal transplantation	30d	0.2	6 months	Healed	No obvious complications
D13	Endophthalmitis	Male /58	Plant-related injury	N	HM	Pars plana vitrectomy+ intravitreal injection of voriconazole +oral voriconazole	28d	HM	6 months	Healed	No obvious complications
D14	Keratitis	Female /55	Plant-related injury	1/2	HM	Corneal debridement +oral voriconazole + voriconazole eye drops	22d	0.6	6 months	Healed	Corneal leukoma
D15	Keratitis	Male /28	Pepper powder exposure to the eye	Full-thickness	0.2	Voriconazole interlamellar corneal injection + oral voriconazole + voriconazole eye drops	90d	0.4	6 months	Healed	Corneal nebula
D16	Keratitis	Male /46	N	1/2	0.2	Voriconazole interlamellar corneal injection + oral voriconazole + voriconazole eye drops	45d	0.5	6 months	Healed	Corneal nebula
D17	Keratitis	Male /41	N	1/2	HM	Corneal debridement +oral voriconazole + voriconazole eye drops	38d	0.4	6 months	Healed	Corneal leukoma
D18	Keratitis	Female /73	Plant-related injury	1/4	HM	Corneal debridement+ oral voriconazole + voriconazole eye drops	20d	0.4	6 months	Healed	Corneal nebula
D19	Keratitis	Male /53	Plant-related injury	2/3	FC	Corneal debridement+ oral voriconazole + voriconazole eye drops	58d	0.2	6 months	Healed	Corneal nebula
D20	Keratitis	Male /45	Allergic conjunctivitis, long-term use of topical steroid eye drops	1/3	0.3	Voriconazole interlamellar corneal injection + oral voriconazole + voriconazole eye drops	33d	0.8	6 months	Healed	Corneal leukoma

BCVA, Best-Corrected Visual Acuity; HM, Hand Movements; FC, Finger Counting; LP, Light Perception.

## Discussion

*P. lilacinum* is a saprophytic filamentous fungus that is widely distributed in soil, fruits and other plants (Luangsa-Ard et al., 2011). This fungus was previously regarded as a contaminant; however, research reports in recent years have indicated an increasing trend in primary fungal infection cases caused by it in humans and animals. Keratitis represents its most common clinical manifestation (Ali et al., 2017; Kuthan et al., 2021; Monden et al., 2012), followed by cutaneous and subcutaneous tissue infections, with cases involving other anatomical sites being relatively rare.

Notably, existing literature reports have identified that the most common predisposing factors for *P. lilacinum* keratitis and endophthalmitis include intra-ocular lens implantation (32.8%), non-surgical trauma with or without a foreign body (20%), ophthalmic surgery (10%), and the wearing of contact lenses(3.3%) (Pastor and Guarro, 2006). In contrast to the findings of the aforementioned studies, plant-related ocular trauma was the primary predisposing factor for *P. lilacinum* keratitis/endophthalmitis in the present study. A total of 12 cases of *P. lilacinum* keratitis/endophthalmitis induced by plant-related trauma were confirmed, accounting for 60% of all cases. Recent studies have demonstrated that the conidial suspension of *P. lilacinum* exhibits nematicidal activity, thus rendering this fungus a useful biocontrol agent (Goffré and Folgarait, 2015). Additionally, *P. lilacinum* is pathogenic to insects, making it a potential biological agent for the control of greenhouse insect and mite pests (Fiedler and Sosnowska, 2007). Given the extensive application of *P. lilacinum* in the agricultural sector, the infected patients in this study were mostly agricultural workers or outdoor laborers, who are frequently exposed to accidental plant-related ocular trauma during agricultural harvesting activities.

The pathogenicity of *P. lilacinum* is attributed to the invasion of the filamentous fungus into injured or diseased ocular surfaces (Juyal et al., 2018), typically caused by fungal fragments or spores embedded in the corneal surface; the use of corticosteroids and immunosuppressants may promote corneal infections (McLintock et al., 2013). *P. lilacinum* exhibits a notable tropism for ocular structures. Invasion of the cornea by *P. lilacinum* induces the production of hydrolytic enzymes that release mycotoxic peptides, which contribute to the inflammatory response in affected tissues (Mikami et al., 1984). Unlike many other filamentous fungi, *P. lilacinum* can germinate in tissues and produce large quantities of conidia, which may explain the propensity of this species to disseminate within the human body (Gordon and Norton, 1985; Orth et al., 1996).

Diagnosis of *P. lilacinum* infections is primarily based on morphological characteristics and histological examination. Confocal microscopy, corneal scraping staining, and histological sections may lead to misidentification of *P. lilacinum* spores as yeast, potentially resulting in misdiagnosis and inappropriate treatment (Liu et al., 1998). In this study, positive confocal microscopy findings (e.g., detection of typical fungal hyphae) showed extremely high specificity (exceeding 90%) for the diagnosis of fungal keratitis, and can thus be used as a reliable indicator for early initiation of antifungal treatment. However, its sensitivity is not 100%, and a negative result cannot completely rule out fungal infection. This is particularly true when hyphae are located in scanning blind areas (e.g., the corneal periphery, the deepest corneal layer) or obscured by dense inflammatory exudates, where filamentous images tend to be undetectable.

Culture remains the gold standard for identifying *P. lilacinum*. This fungus grows rapidly at 28 °C, forming vinaceous to violet-colored colonies after sporulation on Sabouraud dextrose agar (SDA) and PDA. Microscopic examination reveals hyaline hyphae, smooth-walled conidiophores with verticillate branches, and phialides that are swollen at the base and gradually taper into a slender neck. Based on the unique colony color and brush-like conidiophores, the strain can be easily identified morphologically. Given its intrinsic resistance to amphotericin B and natamycin, morphological identification is particularly important for laboratories unable to perform MALDI-TOF MS or molecular sequencing. Molecular methods, such as internal transcribed spacer (ITS) sequence analysis and real-time polymerase chain reaction (PCR), provide effective alternatives for rapid diagnostic testing (Atkins et al., 2005; Houbraken et al., 2010).

The prognosis of *P. lilacinum* corneal infections is generally poor due to the fungus’s high drug resistance, and blindness is not uncommon. *P. lilacinum* has been reported to be resistant to commonly used antifungal agents such as amphotericin B and natamycin (Gordon and Norton, 1985). Although it is usually resistant to fluconazole, it has shown sensitivity to triazole drugs such as itraconazole (Aguilar et al., 1998). In our study, *P. lilacinum* exhibited high MICs or MECs to amphotericin B, 5-fluorouracil, micafungin, caspofungin, and fluconazole; however, it showed low MICs to voriconazole, posaconazole, isavuconazole, and itraconazole. Voriconazole has excellent oral bioavailability and, like other azoles, can reach therapeutic concentrations in the cornea, enabling successful treatment of *P. lilacinum* keratitis. Thus, it represents a viable option when other antifungal therapies yield poor results (Anderson et al., 2004; Ford et al., 2008; Gracitelli et al., 2019). Almeida Oliveira et al. reported successful treatment of refractory *P. lilacinum* keratitis using posaconazole (Almeida Oliveira et al., 2019). While triazole drugs are highly effective against *P. lilacinum*, clinicians face challenges in selecting the appropriate antifungal agent and route of administration. The corneal epithelium acts as a barrier to the penetration of most topical antifungal agents. In our study, we adopted a combination of systemic and topical antifungal drugs for treatment. Corneal scraping to remove the epithelium, necrotic tissue, and mucus can enhance drug penetration. Additionally, intrastromal injection and anterior chamber injection can improve drug permeability. Research has shown that the combined use of systemic and topical voriconazole plus intracameral injection successfully cured recurrent *P. lilacinum* keratitis. This study administered individualized treatments to 20 patients: for endophthalmitis cases, pars plana vitrectomy was performed in combination with intravitreal voriconazole infusion; for corneal ulcer cases, anterior chamber irrigation, corneal debridement combined with medication, intrastromal voriconazole injection, or conjunctival flap covering was adopted, respectively. All cases were additionally treated with oral voriconazole (200 mg twice daily) and topical voriconazole eye drops. The treatment course lasted 20–96 days. Three eyes retained the eyeball but only had HM visual acuity. Most other cases were effectively controlled (with the formation of corneal macula/leukoma), with BCVA ranging from LogMAR 0.2 to 0.8. Four cases underwent corneal transplantation due to poor disease control. These findings indicate that individualized voriconazole-based combination therapy exerts favorable efficacy in controlling the progression of related ophthalmic diseases.

## Conclusion

Early diagnosis and appropriate treatment can result in functional, even good, visual outcomes for patients with *P. lilacinum* keratitis. At our institution, voriconazole is the first-line treatment for suspected or confirmed *P. lilacinum* keratitis. We believe that early initiation of voriconazole therapy, combined with well-planned surgical intervention when necessary, can preserve good visual function while avoiding corneal transplantation and evisceration.

## Data Availability

The raw data supporting the conclusions of this article will be made available by the authors, without undue reservation.
