# Supplementary material for: Clinical characteristics, treatment, and outcomes of fungal keratitis/endophthalmitis caused by Purpureocillium lilacinum
Source: Front Cell Infect Microbiol. 2026 Jun 1;16:1805428. doi: 10.3389/fcimb.2026.1805428 (PMC13265360; doi:10.3389/fcimb.2026.1805428)
Supplement: Supplementary file 1 [file Table1.docx]

Supplementary Table 1 Sequences reference used in this study

| CBS No | Species | ITS Gene Bank Accession N◦^[7]^ |
| --- | --- | --- |
| CBS 129474 | Purpureocillium lilacinum | MH865347.1 |
| CBS 346.51 | Purpureocillium lilacinum | MH856891.1 |
| CBS 226.73B | Purpureocillium lilacinum | MH860675.1 |
| CBS 128677 | Purpureocillium lavendulum | MH864976.1 |
| CBS 372.70 | Paecilomyces maximus | MH859719.1 |
| CBS 371.70 | Paecilomyces maximus | MH859718.1 |
| CBS 339.51 | Paecilomyces variotii | MH856887.1 |
| CBS 338.51 | Paecilomyces variotii | MH856886.1 |
| CBS 284.48 | Paecilomyces divaricatus | MH856344.1 |
| CBS 368.70 | Paecilomyces dactylethromorphus | MH859715.1 |
